# Molecular detection of spotted fever group rickettsiae in hedgehogs (*Erinaceus* *amurensis*) and hedgehog-attached ticks in Xuyi County, Southeast China

**DOI:** 10.1007/s10493-022-00721-y

**Published:** 2022-09-12

**Authors:** Changqiang Zhu, Lele Ai, Yong Qi, Yunsheng Liu, Hong Li, Fuqiang Ye, Qiuwei Wang, Yizhe Luo, Weilong Tan, Chunmeng Shi

**Affiliations:** 1 Institute of Rocket Force Medicine, State Key Laboratory of Trauma, Burns and Combined Injury, Army Medical University, Chongqing, 400038 China; 2 Centre for Diseases Prevention and Control of Eastern Theater, Nanjing, 210002 China

**Keywords:** Hedgehog, Tick, *Rickettsia heilongjiangensis*, *Candidatus* Rickettsia xuyiensis, China

## Abstract

Tick-borne diseases like *Rickettsia*, *Anaplasma* and *Ehrlichia* are widespread infectious zoonoses that threaten the health of both humans and animals worldwide. Ticks and their hosts, such as hedgehogs, can play a crucial role in transmitting tick-borne diseases and the cycle of *Rickettsia*. To investigate the presence and identity of *Rickettsia* in hedgehogs and hedgehog-attached ticks in Xuyi County, Southeast China, 114 ticks were collected from 45 hedgehogs captured totally. Via morphological and molecular methods, all these ticks were identified as two species: *Haemaphysalis flava* (110/114, 96.5%) and *Haemaphysalis longicornis* (4/114, 3.5%). *Rickettsia* spp. were genotypically characterized by PCR targeting *rrs*, *gltA*, *ompA*, *ompB*, and *sca4* gene fragments. The prevalence of spotted fever group rickettsiae (SFGR) infection found in hedgehogs and ticks was 17.8% (8/45) and 78.1% (89/114), respectively. Phylogenetic analyses demonstrated that those *Rickettsia* spp. belong to two species: *Rickettsia heilongjiangensis* (*R. heilongjiangensis* XY-1) and a potential new species, *Candidatus* Rickettsia xuyiensis XY-2. The present study gave the first evidence of *R. heilongjiangensis* and *Candidatus* R. xuyiensis in ticks and hedgehogs of Southeast China. Our findings suggest that hedgehogs might be involved in the natural transmission cycle of *Rickettsia* species.

**Supplementary Information:**

The online version contains supplementary material available at 10.1007/s10493-022-00721-y.

## Introduction

Tick-borne intracellular bacteria, including *Coxiella burnetti*, *Anaplasma* spp., *Ehrlichia* spp. and *Rickettsia* spp., cause emergent or re-emergent infectious diseases among all continents (Ben Said et al. 2018; Boulanger et al. 2019; Eisen 2018; Fang et al. 2021). The genus *Rickettsia* (family Rickettsiaceae, order Rickettsiales) comprise tiny obligate intracellular bacteria capable of infecting humans and animals with mild to severe symptoms (Merhej et al. 2014; Kho et al. 2019; Shpynov et al. 2018).

Ticks are the primary vector and reservoir of *Rickettsia*. The spotted fever group rickettsiae (SFGR), including pathogenic and nonpathogenic species found worldwide, are transmitted mainly by hard ticks (Ixodidae) to vertebrate hosts (Parola et al. 2000; Socolovschi et al. 2009). In China, > 110 species of hard ticks have been identified, of which *Haemaphysalis longicornis* and *H. flava* are the most common species throughout China (Zhang et al. 2019). A variety of *Rickettsia* species—including *R. japonica*, *R. heilongjiangensis*, *R. raoultii*, *Candidatus* Rickettsia tarasevichiae and *Candidatus* Rickettsia principis—have been screened out from *H. longicornis* and *H. flava* (Fang et al. 2021; Jiang et al. 2018; Liu et al. 2016).

Hedgehogs mainly inhabit natural open and green spaces as well as artificial, rural and urban areas, including farmlands, parks, gardens, scrubby habitats at the edge of forests, and shrubby vegetation. They feed on a broad spectrum, including caterpillars, earthworms, small vertebrates, bird eggs, and berries and fruits (Reuter et al. 2019). Hedgehogs are crucial wild animal hosts for various ticks, including *Ixodes hexagonus*, *H. flava*, *H. longicornis*, *H. erinacei*, *H. aegyptium*, *H. marginatum* and *Rhipicephalus sanguineus* (Jahfari et al. 2017; Khaldi et al. 2012; Marié et al. 2012; Szekeres et al. 2019; Orkun et al. 2019; Barradas et al. 2021). Hedgehogs’ ecological and feeding habits, along with high population densities, resulting in their frequent contact with either human or domestic and wild animals, implicates the possibility of tick-borne diseases (Delogu et al. 2020). Therefore, hedgehogs may be involved in the ecology of several potential emerging pathogens.

A wide range of tick-borne bacteria has been reported in hedgehogs and their attached ticks (Skuballa et al. 2010; Szekeres et al. 2019; Bolanos-Rivero et al. 2017; Gong et al. 2020). Therefore, from a public health perspective it is of great importance to understand the local tick species, tick hosts, and SFG rickettsiae carried by them. Despite the previous extensive efforts of clarifying this problem, the knowledge about the circulation of SFG rickettsiae in areas of Southeast China, such as Xuyi County, Jiangsu Province, is still unclear (Jiang et al. 2010; Tan et al. 2012; Li et al. 2018a, b). Therefore, to evaluate the prevalence of SFG rickettsiae within the Southeast China region, the present study collected free-ranging hedgehogs and ticks from Xuyi County, Southeast China, and investigated their diversity and related SFG rickettsiae, in order to provide a scientific basis for the prevention and control of SFGR*.*

## Materials and methods

### Ethical approval

All procedures and protocols for sample collection and processing were approved by the Administrative Committee on Animal Welfare of the Institute of Jiangsu CDC Veterinary and the Ethics Committee of the CDC of Eastern Theater (approval nrs. 2017011 and 2018012; approval dates 26-10-2017 and 15-08-2018).

### Ticks and animal collection

**Fig. 1:**
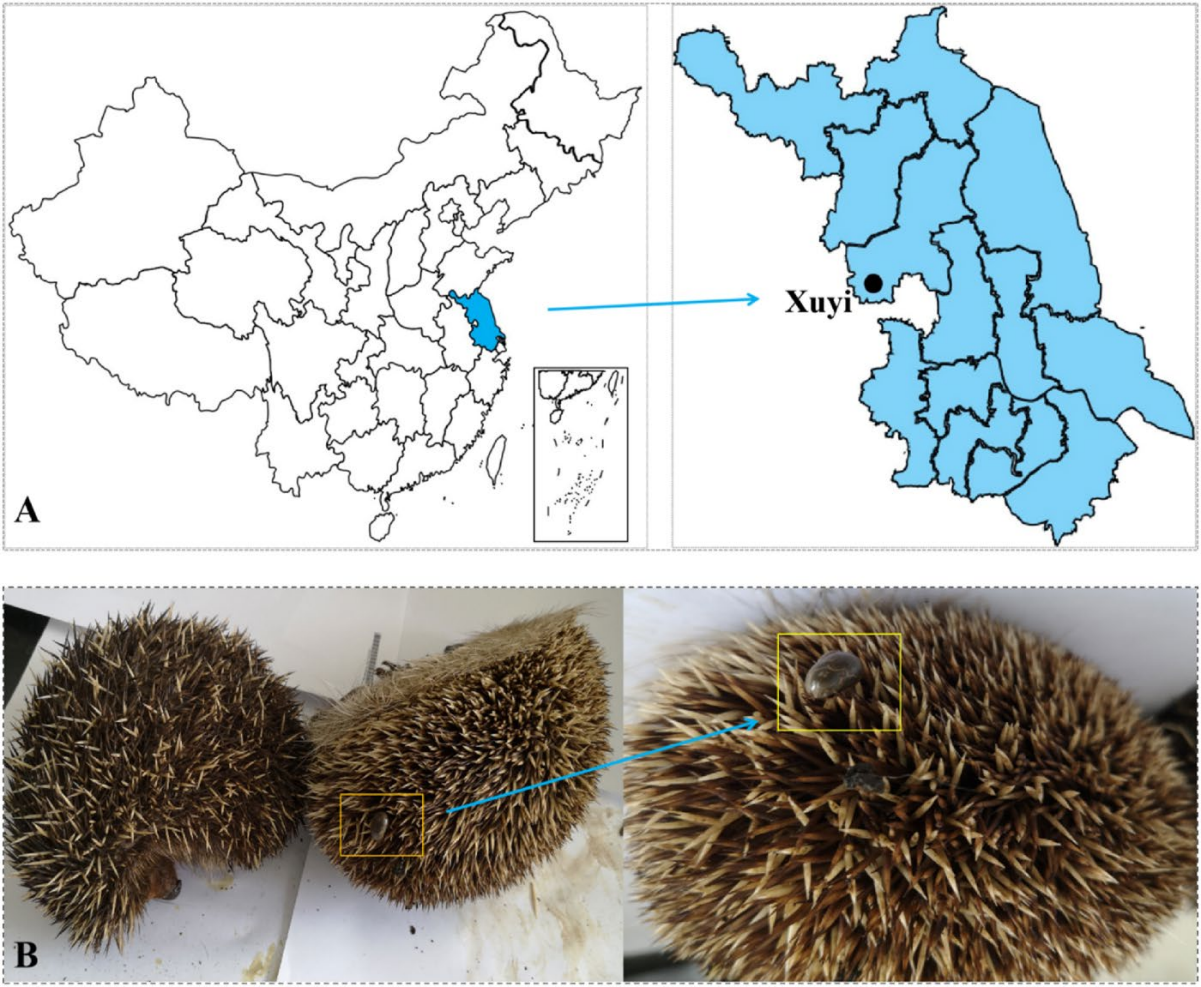
**A** Geographic location of rural area of Xuyi County, Jiangsu Province, where hedgehogs were collected. **B** Two of the Amur hedgehogs collected in Xuyi (●) in the study

Between November 2017 and April 2019, 45 hedgehogs were captured from forest sites near Tieshan Temple in Xuyi County, Jiangsu Province, China (Fig. 1). After careful examination, ectoparasitic ticks were removed from the hedgehogs using fine forceps and placed individually into 1.5-mL tubes with 70% cleaning ethanol. After cleaning, all ticks were marked with the collection date and stored at −80 °C. The tick species were identified based on morphological criteria (Deng and Jiang 1991) and molecular biology tools (Liu et al. 2016). After being anesthetized with diethyl ether, all hedgehogs were sacrificed to collect muscle tissue, hearts, livers, spleens, lungs, kidneys, brains, and intestines, which were all stored at −80 °C.

### DNA extraction

Ticks and hedgehog tissues were homogenized with a stroke-physiological saline solution individually. Homogenates were centrifuged for 10 min at 1000×*g* and 4 °C, and pellets were collected for DNA extraction. Genomic DNA was extracted from each specimen by using the MiniBEST Universal Genomic DNA Extraction Kit (Takara, Beijing, China) according to the manufacturer’s instructions and subsequently stored at −20 °C before use.

### PCR amplification and sequencing

To identify the species of each hedgehog, a partial sequence of the mitochondrial 16S rRNA gene—approximately 201–211 nucleotides (nt) in length—was PCR-amplified using genomic DNA from hedgehog muscle tissues, based on primers (HedF and HedR) as described by Sarri et al. (2014). To identify the species of each tick, the mitochondrial 16S rRNA gene from the genomic DNA of each tick was PCR-amplified using the forward and reverse primers TickHF and TickHR (Liu et al. 2016).

The rickettsial citrate synthase (*gltA*) gene was chosen as the target for its genus specificity and conservativeness (Mediannikov et al. 2004). All samples were screened for the presence of *gltA* by nested PCR using two sets of primers, RpCS877F and RpCS1258R, and approximately a 380 bp fragment of the *gltA* gene was amplified. The second PCR round will be performed if no product was visible by agarose gel electrophoresis. The full-length of *gltA* gene was amplified in 22 tick samples using primers CS2d and CSEndr. To further characterize SFGR strains, each positive sample for the *gltA* gene was tested for four other genes: the 16S ribosomal RNA gene (16S rRNA), outer membrane protein A gene (*ompA*), outer membrane protein B gene (*ompB*), and surface cell antigen-4 gene (*sca4*). The primer sets used in each of these assays are listed in Table 1. Sterile distilled water and a previously determined rickettsial-positive tick sample were used as negative and positive controls in each run, respectively. All positive amplicons were purified with PCR Clean-Up Kit (Beyotime, Shanghai, China). Sanger dideoxy DNA sequencing was performed using the BigDye Terminator v.3.1 Cycle Sequencing Kit (Applied Biosystems, Foster City, CA, USA) and an ABI Prism 3130 × genetic analyzer.

**Table 1:** Primers used in the present study

Primer name	Sequence (5′–3′)	Target gene	Annealing temperature (°C)	Amplicon size (bp)	References
HedF	AΥAAGACGAGAAGACCC	Hedgehog 16S ribosomal RNA gene (16S rRNA)	53	222–252	Sarri et al. (2014)
HedR	GATTGCGCTGTTATTCC				
TickHF	GGTATTTTGACTATA CAA AGGTAT TG	Tick 16S ribosomal RNA gene (16S rRNA)	54	262–278	Liu et al. (2016)
TickHR	TTATTACGCTGTTATCCCTAGAGTATT				
Rick_16S_F3	ATCAGTACGGAATAACTTTTA	16S ribosomal RNA gene (16S rRNA)	52	1328	Anstead and Chilton (2013)
Rick_16S_F4	TGCCTCTTGCGTTAGCTCAC				
CS2d	ATGACCAATGAAAATAATAAT	Citrate synthase gene (*gltA*)	50	1120	Mediannikov et al. (2004)
CSendR	CTTATACTCTCTATGTACA				
RpCS877p	GGGGACCTGCTCACGGCGG		54	380	
RpCS1258n	ATTGCAAAAAGTACAGTGAACA				
190–70	ATGGCGAATATTTCTCCAAAA	Outer membrane A gene (*ompA*)	53/48	542	Fournier et al. (1998)
190–701	GTTCCGTTAATGGCAGCATCT				
120_2788	AAACAATAATCAAGGTACTGT	Outer membrane B gene (*ompB*)	48	816	Roux et al. (2000)
120_3599	TACTTCCGGTTACAGCAAAGT				
D1f	ATGAGTAAAGACGGTAACCT	Surface cell antigen-4 (*sca4*)	50	928	Sekeyova et al. (2001)
D928r	AAGCTATTGCGTCATCTCCG				

### Phylogenetic analysis

Partial nucleotide sequences of *rrs*, *gltA*, *ompA*, *ompB*, and *sca4* obtained from ticks, and hedgehog organs were compared to known sequences using the BLAST program from the NCBI website (https://blast.ncbi.nlm.nih.gov/Blast.cgi). The best-fit evolution model for each gene was calculated via MrModeltest v.2.3 in cooperation with BEAST v.1.10.4 using the Bayesian information criterion (BIC). The K81u (K3Pu), TVM, TPM3u, TN(TN93) and K81u (K3Pu) models were selected for the *gltA*, *ompA*, *ompB*, *rrs*, and *sca4* gene, respectively. Substitution rates at polymorphic sites in both genes followed a gamma distribution with a large proportion of invariable sites. Maximum Likelihood (ML) methods of phylogeny inference on the individual were conducted in BEAST in the ‘Ultrafast’ bootstrap model with the 5000 bootstrap samples, maximum interaction value 1000, and minimum correlation coefficient of 0.90. Nodal support was evaluated by bootstrap resampling for the ML trees from posterior probabilities (PP) for Bayesian inferences. Bootstrap values of 70% or more and Bayesian support values of 0.95 and higher were considered significant nodal support.

## Results

The amplified partial sequences (203–215 nt) of the hosts’ 16S rRNA gene (GenBank acc. nr. of the hedgehogs: OM865773) showed the highest nucleotide similarity (96.0–97.0%) to those of *Erinaceus amurensis* from the GenBank database (KX9646061). Therefore, all hedgehogs (n = 45) were identified as *E. amurensis*. 17.8% (8/45) of the hedgehogs were PCR positive for rickettsial *gltA*. The highest infection rate of SFGR in the eight positive hedgehogs was seen in the brain, whereas other organs varied in their presence of rickettsial *gltA* (Table 2).

**Table 2:** *Rickettsia* spp. detection in hedgehogs and *Haemaphysalis flava* and *H. longicornis* (*H. long.*) collected from 45 hedgehogs from Xuyi, 2017–2019

Hedgehog no. (collection date/gender)	Season of hedgehog collection	Tick species and sex				*H. flava* (no. positives/tested)	*H. longicornis* (no. positives/tested)	Hedgehog organs (no. positives/tested)							*Rickettsia* spp.
		*H. flava*		*H. long.*											
		F	M	F	M			a	b	c	d	e	f	g	
1 (2017/F)	Winter	1	−	−	−	0/1	−	−	−	−	−	−	−	−	
2 (2017/M)	Winter	3	−	−	−	2/3	−	−	−	−	−	−	−	−	*R. heilongjiangensis*
3 (2017 M)	Winter	−	−	−	−	−	−	−	−	−	−	−	−	−	
4 (2017/M)	Winter	−	−	−	−	−	−	−	−	−	−	−	−	−	
5 (2017/F)	Winter	−	−	−	−	−	−	−	−	−	−	−	−	−	
6 (2017/M)	Winter	−	−	−	−	−	−	−	−	−	−	−	−	−	
7 (2017/F)	Winter	4	1	−	2	3/5	0/2	−	−	−	−	−	−	−	*R. heilongjiangensis*
8 (2017/M)	Winter	−	−	−	−	−	−	−	−	−	−	−	−	−	
9 (2017/M)	Winter	−	1	−	−	1/1	−	−	−	−	−	−	−	−	*R. heilongjiangensis*
10 (2018/M)	Spring	−	−	−	−	−	−	−	−	−	−	−	−	−	
11 (2018/F)	Spring	−	−	−	−	−	−	−	−	−	−	−	−	−	
12 (2018/M)	Spring	−	−	−	−	−	−	−	−	−	−	−	−	−	
13 (2018/M)	Spring	3	−	−	−	2/3	−	−	−	−	−	−	−	−	*R. heilongjiangensis*
14 (2018/M)	Spring	−	−	−	−	−	−	−	−	−	−	−	−	−	
15 (2018/M)	Summer	−	−	−	−	−	−	−	−	−	−	−	−	−	
16 (2018/F)	Summer	5	1	−	−	4/6	−	−	+	−	−	−	−	−	*R. heilongjiangensis*
17 (2018/F)	Summer	4		−	−	0/4	−	−	−	−	−	−	−	−	
18 (2018/M)	Summer	1	7	−	2	7/8	0/2	−	−	−	−	−	−	−	*R. heilongjiangensis*
19 (2018/M)	Autumn	−	−	−	−	−	−	−	−	−	−	−	−	−	
20 (2018/F)	Autumn	−	3	−	−	1/3	−	−	−	−	−	−	−	−	*R. heilongjiangensis*
21 (2018/M)	Autumn	−	−	−	−	−	−	−	−	−	−	−	−	−	
22 (2018/M)	Autumn	1	10	−	−	10/11	−	−	−	+	−	−	−	−	*R. heilongjiangensis*
23 (2018/F)	Autumn	5	3	−	−	8/8	−	−	−	−	−	−	−	+	Candidatus *Rickettsia*
24 (2018/M)	Autumn	−	−	−	−	−	−	−	−	−	−	−	−	−	
25 (2019/M)	Spring	4	7	−	−	9/11	−	−	+	−	−	−	−	−	*R. heilongjiangensis*
26 (2019/F)	Spring	1	5	−	−	6/6	−	−	−	−	+	−	+	−	*R. heilongjiangensis*
27 (2019/M)	Spring	−	−	−	−	−	−	−	−	−	−	−	−	−	
28 (2019/M)	Spring	−	−	−	−	−	−	−	−	−	−	−	−	−	
29 (2019/F)	Spring	−	3	−	−	3/3	−	−	−	−	−	−	−	−	*R. heilongjiangensis*
30 (2019/M)	Spring	−	−	−	−	−	−	−	−	−	−	−	−	−	
31 (2019/F)	Summer	−	−	−	−	−	−	−	−	−	−	−	−	−	
32 (2019/M)	Summer	1	4	−	−	5/5	−	−	−	−	−	−	−	−	Candidatus *Rickettsia*
33 (2019/M)	Summer	−	−	−	−	−	−	−	−	−	−	−	−	−	
34 (2019/F)	Summer	−	−	−	−	−	−	−	−	−	−	−	−	−	
35 (2019/M)	Autumn	3	12	−	−	14/15	−	−	+	−	−	+	+	−	*R. heilongjiangensis*
36 (2019/M)	Autumn	−	5	−	−	4/5	−	+	+	−	−	−	+	−	*R. heilongjiangensis*
37 (2019/F)	Autumn	−	6	−	−	5/6	−	−	+	−	−	−	−	−	*R. heilongjiangensis*
38 (2019/F)	Autumn	−	−	−	−	−	−	−	−	−	−	−	−	−	
39 (2019/M)	Autumn	2	−	−	−	1/2	−	−	−	−	−	−	−	−	*R. heilongjiangensis*
40 (2019/M)	Winter	−	4	−	−	2/4	−	−	−	−	−	−	−	−	*R. heilongjiangensis*
41 (2019/F)	Winter	−	−	−	−	−	−	−	−	−	−	−	−	−	
42 (2019/M)	Winter			−	−		−	−	−	−	−	−	−	−	*R. heilongjiangensis*
43 (2019/F)	Winter	−	−	−	−	−	−	−	−	−	−	−	−	−	
44 (2019/F)	Winter	−	−	−	−	−	−	−	−	−	−	−	−	−	
45 (2019/M)	Winter	−	−	−	−	−	−	−	−	−	−	−	−	−	
Total		110		4		87/110	0/4	1	5	1	1	1	3	1	

Tick sex: F, female; M, male. Hedgehog organs: a, heart; b, brain; c, intestine; d, spleen; e, lung; f, liver; g, kidney

A total of 114 adult ticks were collected from 45 hedgehogs and identified as *H. flava* and *H. longicornis* through their morphological characteristics and partial 16S rRNA gene (Table 2). 110 of the 114 tested *Haemaphysalis* ticks (96.5%, GenBank acc. nr. MH520707.1) were identified as *H. flava* (GenBank acc. nr. OM865774), with a similarity of 99.2%. The other four (3.5%, GenBank acc. nr. KX083342.1) were identified as *H. longicornis* (GenBank acc. nr. OM865775), with a likeness of 99.2%. Overall, 89 of 114 ticks (78.1%) were tested positive for SFGR, with the infection rate as 80.9% (89/110) in *H. flava* and 0% (0/4) in *H. longicornis*, respectively.

**Fig. 2:**
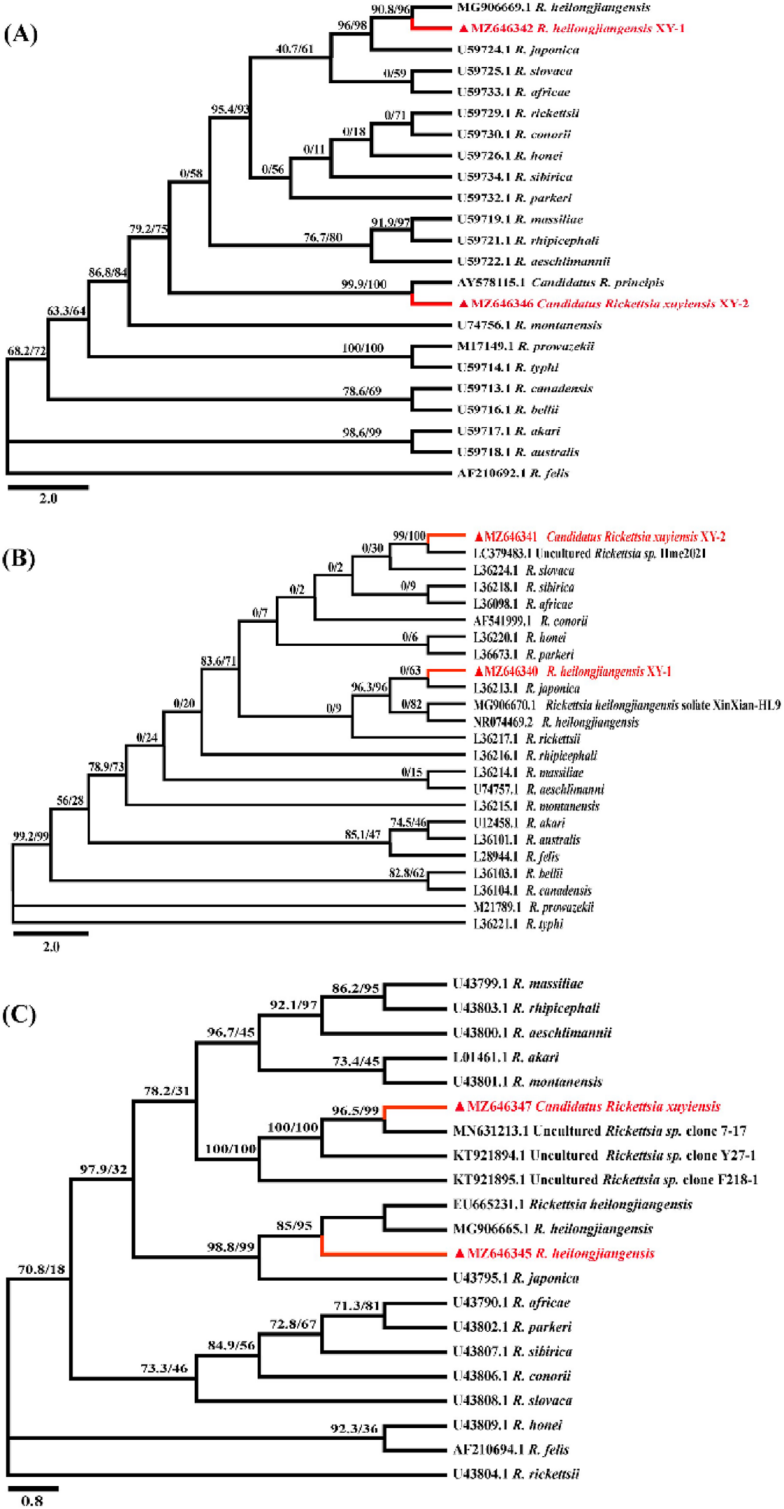

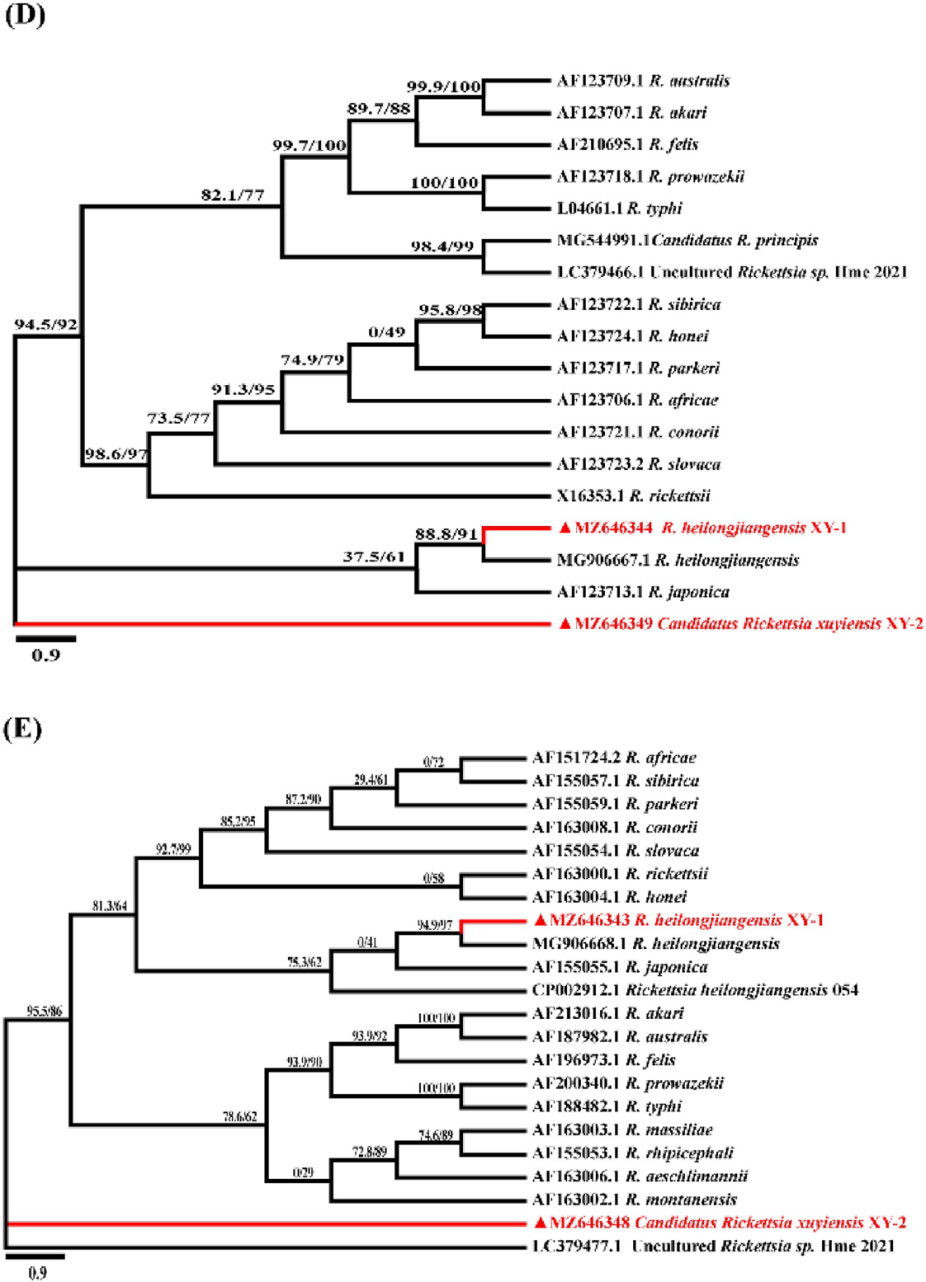
Phylogenetic tree of *Rickettsia* spp. detected in ticks and hedgehogs from Southeast China with other rickettsial strains based on partial (**A**) *rrs*, (**B**) *gltA*, (**C**) *ompA*, (**D**) *ompB* and (**E**) *sca4* sequences. Rickettsial strains in red () were detected in ticks and hedgehogs of this study. Maximum likelihood (ML) methods of phylogeny inference on individuals were conducted in BEAST v.1.10.4 under the ‘Ultrafast’ Bootstrap model with 5000 bootstrap samples, maximum interaction value 1000 and minimum correlation coefficient of 0.90

An 1183 bp *rrs*, 1153 bp *gltA*, 602 bp *ompA*, 784 bp *ompB*, and 861 bp *sca4* gene fragment of *Rickettsia* spp. was amplified and sequenced from our partial positive samples, which showed 100% identity to *R. heilongjiangensis* isolate Xinxian-HL9 (China), with the GenBank acc. nrs. MG9066701, MG9066691, MG9066651, MG9066671 and MG9066681, respectively. Meanwhile, phylogenetic trees (Fig. 2A–E, respectively), inferred from these genes, also showed one isolated strain formed a distinct cluster with *R. heilongjiangensis* in all trees, which also confirmed the identification of *R. heilongjiangensis* (*Rickettsia heilongjiangensis* XY-1).

Another 1230 bp *rrs*, 1156 bp *gltA*, 540 bp *ompA*, 789 bp *ompB*, and 886 bp *sca4* gene fragment of *Rickettsia* spp. was amplified and sequenced from the positive ticks. The *rrs* sequence showed 99.7% nucleotide identity with *Candidatus* Rickettsia principis (MG5172531), the *gltA* sequence showed 99.8% nucleotide identity with *Candidatus* R. principis (AY5781151), the *ompB* sequence showed 96.9% nucleotide identity with *Candidatus* R. principis isolate (MG5449911), *ompA* sequence showed 98.6% nucleotide identity with *Rickettsia* sp. NGT116-2016-Hfla (LC4610751) and sca4 showed 99.2% nucleotide identity with an uncultured *Rickettsia* sp. Hme_2021 (LC3794771). The isolated strain could not be classified into specific species due to a lack of consensus between the phylogenetic trees (Fig. 2). According to the gene sequence-based criteria proposed by Fournier et al. (2003), this *Rickettsia* isolate can therefore be classified as a potentially novel SFGR, named *Candidatus* Rickettsia xuyiensis-XY2.

GenBank acc. nrs. of partial sequences obtained in the study are: MZ646340-MZ646341 (*rrs* genes of *Rickettsia* spp*.*), MZ646342-MZ646345 (*gltA*, *sca4*, *ompB*, and *ompA* gene of *R. heilongjiangensis* XY-1), MZ646346-MZ646349 (*gltA*, *ompA*, *sca4*, and *ompB* gene of *Candidatus* R. xuyiensis XY-2), OM865773(16S rRNA gene of hedgehog), OM865774 and OM865775 (16S rRNA gene of *H. flava* and *H. longicornis*).

## Discussion

This study reports *Rickettsia* sp.'s finding in hedgehogs and ticks from Jiangsu Province, Southeast China. In addition, this is the first report of *R. heilongjiangensis* and a novel potential species of *Rickettsia* (*Candidatus* R. xuyiensis XY-2) in *H. flava* and hedgehogs of Xuyi, Southeast China.

SFGRs are widely distributed throughout China and tend to have regional characteristics. Previous studies have revealed the extensive diversity of rickettsiae among tick species and geographic areas (El-Mahallawy et al. 2015; Fang et al. 2021). There are many hills and low mountains in the Xuyi area, and it has a developed presence of animal husbandry. Many tick bites have been reported among fever cases with thrombocytopenia syndrome in Jiangsu Province (Li et al. 2017). Therefore, improving the knowledge on the prevalence of *Rickettsia* in ticks and hosts from this region can identify potential rickettsioses in the population and reduce the risk for tick-borne *Rickettsia* transmission.

Small mammals and ticks are intermediate hosts or vectors of many zoonoses. Hedgehogs, one of the most important hosts of ticks, can play an essential role in the natural foci of tick-borne pathogens (Orkun et al. 2019). Our results demonstrate that the dominant tick species carried by hedgehogs in the Xuyi area is *H. flava*, followed by *H. longicornis*, which agrees with the findings of both Sun et al. (2009) and Lan et al. (2016). The number of ticks carried by each hedgehog in this study may vary significantly due to the sampling season or the activity tracking of hedgehogs. Additionally, an initial screening test using *gltA* nested PCR revealed that 78.1% of the ticks and 17.8% of the hedgehogs were infected with SFG rickettsiae. This percentage was significantly higher than previous work from the Sichuan (33.5%), Yunnan (12.1%), and Zhejiang (7.5%) provinces (Liu et al. 2020; Sun et al. 2015; Zhang et al. 2018). We found that the prevalence of SFGR infection of ticks in eight positive hedgehogs was 76.9 ~ 100%, and most SFGR infection of ticks collected from negative hedgehogs was 0–100%. Of 89 ticks infected with SFG rickettsiae, 63 positive ticks were carried by eight positive hedgehogs (Table 2). As the ticks may suck the blood of these hedgehogs, they had a high positive rate of spotted fever.

Furthermore, we determined partial sequences of the *gltA* gene of SFG rickettsiae by conventional PCR in 45 hedgehogs organs (heart, brain, intestine, spleen, lung, liver, kidney). Based on the sequences of the *gltA* gene obtained from 45 hedgehogs’ organs, the highest infection rate of SFG rickettsiae was detected in the brain (5/45); other organs varied in their presence of rickettsial *gltA* (Table 2). Therefore, the brain of the hedgehog may be particularly susceptible to *Rickettsia.* Our findings also indicate that hedgehogs and their carrying ticks can serve as the animal host and vector for SFG rickettsiae.

For the molecular classification of SFG rickettsiae that were obtained in the study, partial sequences of *rrs*, *gltA*, *ompA*, *ompB*, and *sca4* were analyzed. Phylogenetic trees inferred from *rrs*, *gltA*, *ompA*, *ompB*, and *sca4* analysis are shown in Fig. 2. One isolated strain formed a distinct cluster with *R. heilongjiangensis* in all trees (Fig. 2) and thus were identified as *R. heilongjiangensis* (XY-1). Nucleotide sequence analysis of five genes of *R. heilongjiangensis* XY-1 showed 97–100% similarity with *R. heilongjiangensis* isolate Xinxian-HL9 and *R. japonica* YH_M. The isolated strain could not be classified into specific species due to a lack of consensus between the phylogenetic trees. It shares a branch with the previously reported *Candidatus* R. principis isolate Kh-81, uncultured *Rickettsia* sp. Hme_2021, uncultured *Rickettsia* sp. clone 7–17, whereas it forms a separate branch in the *ompB* and *sca4* phylogenetic tree (Fig. 2). Moreover, in the isolated strain, the sequence nucleotide identity to recognized *Rickettsia* species was < 99.8, 99.9, 98.8, 99.2 and 99.3% for *rrs*, *gltA*, *ompA*, *ompB*, and *sca4*, respectively, which suggests that this agent is novel potential SFG *Rickettsia* according to Fournier et al. (2003)*.* Therefore, this species was provisionally named *Candidatus* R. xuyiensis-XY2, concerning the location where it was found. Our findings indicate that hedgehogs and *H. flava* collected from hedgehogs in Southeast China were infected with *R. heilongjiangensis* and *Candidatus* R. xuyiensis. The diseases caused by these pathogens should therefore be monitored in Southeast China. Further isolation and identification are needed to obtain morphological characteristics and the entire genome of these species. Previous studies have reported that *H. longicornis* is an essential vector of *R. heilongjangensis* (Jiang et al. 2019; Liu et al. 2020; Zhuang et al. 2018). However, no rickettsiae were detected in any of the *H. longicornis* ticks collected in our study, which may be due to the limited sample size of *H. longicornis* ticks.

There are some limitations of this study worth noting. Firstly, our investigation is biased because the infection rates were calculated using ticks collected from the infected hedgehogs, where we collected fewer ticks from uninfected hedgehogs. Therefore, the actual infection rates might be lower than those determined by this research. Secondly, we mainly focused on the infection rates and tick species collected from hedgehogs, and we did not identify ticks carried by other small mammals in the Xuyi area. Thus, it is crucial to find Rickettsiales infection among other local animals and humans in a subsequent study.

## Supplementary Information

Below is the link to the electronic supplementary material.

Supplementary file1 (DOCX 24 kb)
