# Supplementary material for: Molecular detection of spotted fever group rickettsiae in hedgehogs (Erinaceus amurensis) and hedgehog-attached ticks in Xuyi County, Southeast China
Source: Exp Appl Acarol. 2022 Sep 12;88(1):97–111. doi: 10.1007/s10493-022-00721-y (PMC9663401; doi:10.1007/s10493-022-00721-y)
Supplement: Supplementary file 1 — Supplementary file1 (DOCX 24 kb) [file 10493_2022_721_MOESM1_ESM.docx]

Supplementary Table 1. Identity of Rickettsia molecular isolates from Hedgehogs, ticks collected from 45 hedgehogs in Xuyi to the closest recognized Rickettsia species by BLASTN search of five genes in GenBank.

| Rickettsia isolate | Gene | rrs | gltA | ompA | ompB | sca4 |
| --- | --- | --- | --- | --- | --- | --- |
| Candidatus Rickettsia xuyiensis XY-2 | Size (bp) | 1230 | 1156 | 540 | 789 | 886 |
|  | % identity to Uncultured Rickettsia sp. Hme_2021 | 97.9 | 99.6 |  | 96.3 | 99.2 |
|  | % identity to Candidatus Rickettsia principis isolate Kh-81 | 99.7 | 99.8 |  | 96.9 |  |
|  | % identity to Uncultured Rickettsia sp. clone Y27-1 |  |  | 95.9 |  |  |
|  | % identity to Candidatus Rickettsia principis douglasi 061 |  | 99.8 |  |  |  |
|  | % identity to Rickettsia sp. NGT116-2016-Hfla |  |  | 98.6 |  |  |
|  | % identity to Rickettsia sp. isolate EEZA-CRETAV |  |  |  |  | 99.29 |
| Rickettsia heilongjiangensis XY-1 | Size (bp) | 1183 | 1153 | 602 | 784 | 861 |
|  | % identity to Rickettsia heilongjiangensis isolate Xinxian-HL9 | 100 | 100 | 100 | 100 | 100 |
|  | % identity to Rickettsia japonica YH_M | 99.9 | 99.7 | 97.0 | 97.7 | 99.3 |
| Fournier et al., 2003 criteria | Genus | > 98.1% | > 86.5% |  |  |  |
|  | Spotted fever group | > 98.8% | > 92.7% |  | > 85.8% | > 82.2% |
|  | New species | < 99.8% | < 99.9% | <98.8% | < 99.2% | < 99.3% |
